# X-Ray Fluorescence Microscopy Reveals Accumulation and Secretion of Discrete Intracellular Zinc Pools in the Lactating Mouse Mammary Gland

**DOI:** 10.1371/journal.pone.0011078

**Published:** 2010-06-11

**Authors:** Nicholas McCormick, Vanessa Velasquez, Lydia Finney, Stefan Vogt, Shannon L. Kelleher

**Affiliations:** 1 Department of Nutritional Sciences, The Pennsylvania State University, University Park, Pennsylvania, United States of America; 2 Argonne National Laboratory, Argonne, Illinois, United States of America; Brunel University, United Kingdom

## Abstract

**Background:**

The mammary gland is responsible for the transfer of a tremendous amount of zinc (∼1–3 mg zinc/day) from maternal circulation into milk during lactation to support the growth and development of the offspring. When this process is compromised, severe zinc deficiency compromises neuronal development and immune function and increases infant morbidity and/or mortality. It remains unclear as to how the lactating mammary gland dynamically integrates zinc import from maternal circulation with the enormous amount of zinc that is secreted into milk.

**Methodology/Principal Findings:**

Herein we utilized X-ray fluorescence microscopy (XFM) which allowed for the visualization and quantification of the process of zinc transfer through the mammary gland of the lactating mouse. Our data illustrate that a large amount of zinc first accumulates in the mammary gland during lactation. Interestingly, this zinc is not cytosolic, but accumulated in large, discrete sub-cellular compartments. These zinc pools were then redistributed to small intracellular vesicles destined for secretion in a prolactin-responsive manner. Confocal microscopy identified mitochondria and the Golgi apparatus as the sub-cellular compartments which accumulate zinc; however, zinc pools in the Golgi apparatus, but not mitochondria are redistributed to vesicles destined for secretion during lactation.

**Conclusions/Significance:**

Our data directly implicate the Golgi apparatus in providing a large, mobilizable zinc storage pool to assist in providing for the tremendous amount of zinc that is secreted into milk. Interestingly, our study also provides compelling evidence that mitochondrial zinc pools expand in the mammary gland during lactation which we speculate may play a role in regulating mammary gland function.

## Introduction

The development of the mammary gland and lactation is the hallmark of the evolution of mammalian species. During lactation, the mammary gland must tightly regulate the accumulation, production and the ultimate secretion of a complex mixture of nutrients and non-nutritive factors into milk to nourish the developing offspring. This complex process is primarily regulated through lactogenic hormone activation [1], [2], [3], [4] of highly specialized, secretory mammary epithelial cells. Mammary epithelial cells surround the lumen of mammary gland alveoli into which milk components are secreted, pooled and eventually released in a hormonally-mediated manner. Regulation of milk component secretion is dependent upon the specific milk constituent. For example, lactose [5] and secreted proteins [6] are synthesized in the Golgi apparatus and transported through the secretory compartment for exocytosis into milk while lipids are extruded from the apical membrane in the form of droplets encased within the milk fat globule membrane [7]. Hormones and growth factors are transcytosed directly across the mammary epithelial cell [6]. Small ions and water [8] may be directly transported across the apical membrane into the alveolar lumen by specific transporters. The mechanisms through which different trace elements such as iron, copper and zinc (Zn) are exported into milk are unique, reflecting the specificity of trace element transport systems [9], [10], [11], [12]. Additionally, the biological complexation of each trace element in milk is different. For example, iron is bound to lactoferrin and/or transferrin in a species-dependent manner [13], [14] while copper is generally bound to ceruloplasmin and metallothionein [15], [16]. In stark contrast, Zn is predominantly found loosely associated with small molecular weight ligands such as citrate [15], [17]. Moreover, milk Zn concentration is ∼10 times greater (∼2 mg/L,) than the concentration of iron or copper in milk (∼0.2 mg/L). This suggests that the mammary gland has developed unique mechanisms to transfer such an extraordinary amount of Zn into milk during lactation.

How the mammary gland facilitates and regulates the transfer of such a large amount of Zn for secretion into milk during lactation is not currently understood. Our previous studies suggest that Zn transfer into milk involves the integration of mammary gland Zn import, Zn sequestration and Zn secretion mechanisms [18], [19] in a hormonally regulated manner. Presumably, Zn acquisition from maternal circulation by the mammary gland must first be increased to provide enough Zn for secretion into milk. In fact, the lactogenic hormone prolactin increases Zn uptake in mammary cells [18], suggesting that hormonal regulation of Zn uptake into the mammary cell is one point of control. Current dogma suggests that Zn import from maternal circulation likely occurs through members of the Zip family (*SLC39A*) of Zn importers. A specific Zip protein(s) responsible for regulating Zn import into the mammary gland from maternal circulation has yet to be elucidated. Following Zn uptake, the mammary epithelial cell must secrete a tremendous amount of loosely bound Zn into milk. Studies suggest that similar to pancreas [20], brain [21], and prostate [22] the mammary gland may utilize a vesicular process to accomplish such bulk and efficient Zn secretion [23]. We postulate that the mammary epithelial cell must first accumulate Zn into intracellular pools and then redistribute these Zn pools to facilitate such a massive amount of Zn secretion in a hormonally-mediated manner. Consistent with this hypothesis, our recent evidence indicates that vesicles containing “labile” Zn (zincosomes) accumulate in cultured mammary epithelial cells prior to secretion from the cell [23]. Additionally, there is evidence that mitochondria may participate in Zn sequestration and redistribution in several highly specialized cell-types. For example, mitochondrial Zn pools in neurons accumulate and are later mobilized in response to exogenous or endogenous cues [24] prior to transfer to specific cytosolic Zn pools [25]. Comparisons are often made between the secreting mammary and prostate glands. Interestingly, Zn levels in mitochondria isolated from lateral prostate are higher (∼20-fold) compared with mitochondria from other soft tissues [26]. High mitochondria Zn levels parallel Zn levels secreted into the alveoli of the lateral prostate [22] suggesting that the source of Zn for secretion into prostate fluid may be Zn previously sequestered in these mitochondrial pools. As a result, mitochondria may provide a “labile” Zn pool in some specialized cell types with unique Zn secretory requirements such as the prostate and mammary glands.

To explore the hypothesis that the lactating mammary gland must first accumulate Zn prior to secretion, we used the mouse mammary gland and cultured normal mouse mammary epithelial cells as models. We first utilized X-ray fluorescence microscopy (XFM) to visualize and quantify the expansion of Zn pools in the lactating mammary gland and explored the redistribution of these Zn pools in response to the lactogenic hormone prolactin *in vivo*. Secondly, we utilized cultured mammary epithelial cells to identify the specific sub-cellular compartments that accumulate Zn which is then redistributed for secretion from the cell. In this report we document that Zn accumulates in two discrete intracellular pools which include the Golgi apparatus and mitochondria. However, unlike observations in neurons and prostate, only Zn pools within the Golgi apparatus appear to be “labile” and thus redistributed to exocytotic vesicles for secretion.

## Results

**Figure 1:**
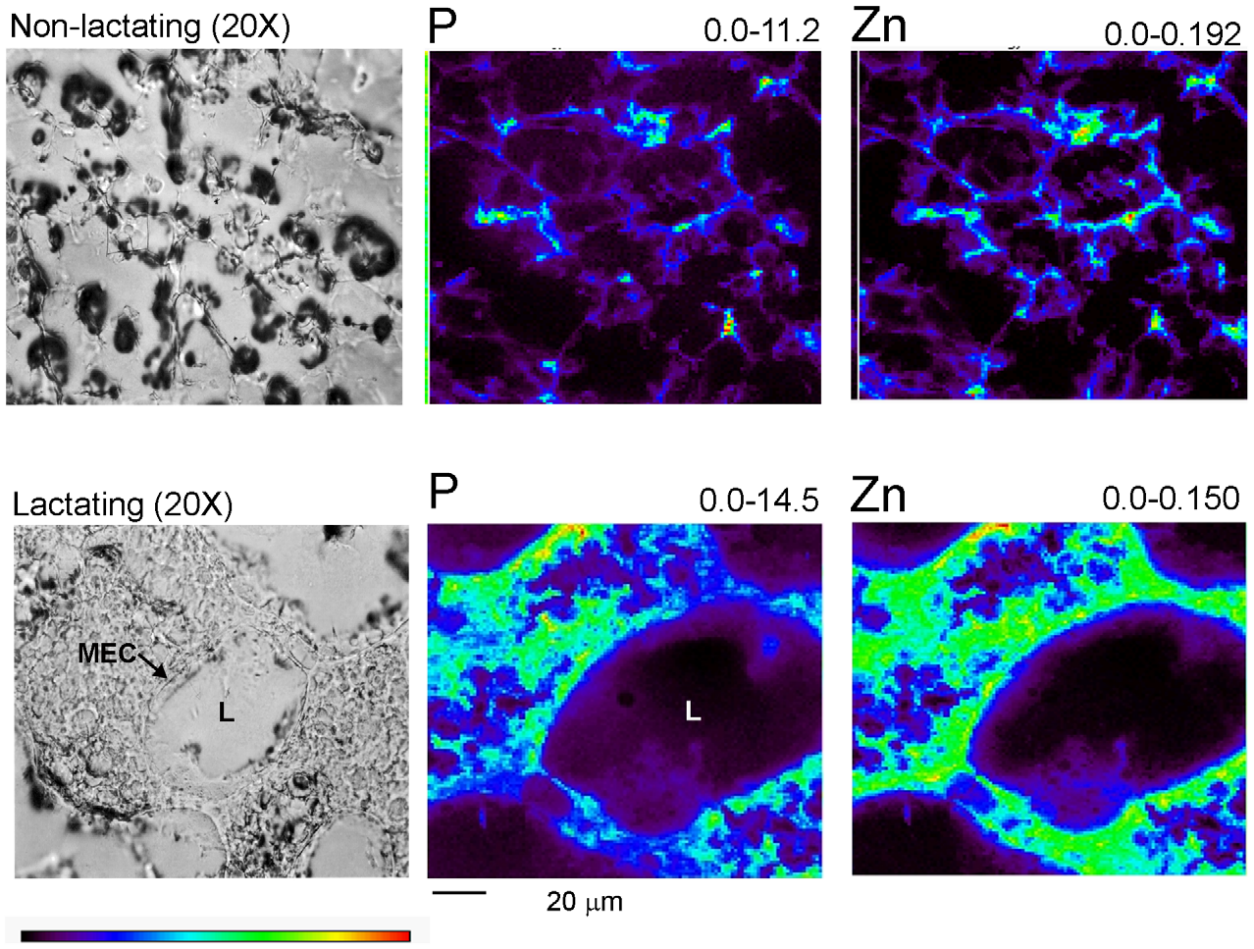
XFM analysis of the mammary gland from non-lactating and lactating mice. Mouse mammary gland (20 µm) was visualized by phase contrast microscopy (20× magnification) and analyzed using XFM. The alveoli lumen (L) is surrounded by a single layer of mammary epithelial cells (MEC). The corresponding element (phosphorus, P; zinc, Zn) and its minimum and maximum threshold values in micrograms per square centimeter are given above each image. The rainbow-colored scale bar reflects the signal intensity measured as micrograms per square centimeter in each pixel, with darker pixels representing areas of low concentration and brighter pixels representing areas of increasing concentration. A scale bar (20 µm) is shown below the elemental maps.

**Figure 2:**
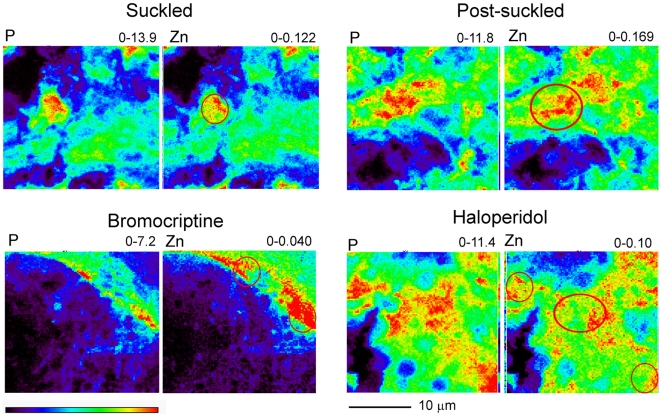
XFM analysis of intracellular Zn pools in mouse mammary gland during lactation. Mouse mammary gland (20 µm) was analyzed using XFM. The corresponding element (phosphorus, P; zinc, Zn) and its minimum and maximum threshold values in micrograms per square centimeter are given above each image. The rainbow-colored scale bar reflects the signal intensity measured as micrograms per square centimeter in each pixel, with darker pixels representing areas of low concentration and brighter pixels representing areas of increasing concentration. A scale bar (10 µm) is shown below the elemental maps. Zinc and phosphorus maps of ∼1–4 mammary cells in the mammary gland from a mouse that had suckled her offspring for 30 min (suckled; used as a baseline), a suckled mouse, 60 min post-suckling (Post-suckled; reflecting Zn accumulation), a mouse injected with bromocriptine (Bromocriptine; used to eliminate prolactin secretion) and a mouse injected with haloperidol (Haloperidol; used to increase prolactin secretion). The red circles on the Zn maps denote corresponding nuclei.

As a model system to test the hypothesis that the mammary gland must first accumulate Zn prior to secretion into milk we compared the concentration and spatial distribution of Zn using XFM. As illustrated in Figure 1, histological features of the mammary gland change dramatically during the transition from a non-lactating to a lactating tissue. Figure 1 illustrates the compact, undifferentiated nature of the non-lactating mammary gland. In contrast, the mammary gland becomes highly differentiated during lactation. There is extensive morphological reorganization into discrete alveoli, surrounded by secretory mammary epithelial cells. These cells surround an open lumen into which milk components are secreted and pooled until removed in response to suckling. Moreover, the mammary gland accumulates a large amount of Zn during lactation to help ensure adequate transfer into milk. To explore the distribution of sub-cellular Zn pools in mammary epithelial cells, we examined the spatial distribution of intracellular Zn pools in lactating mammary gland *in vivo* and in cultured mammary epithelial cells *in vitro* (Figure 2). XFM analysis of mouse mammary gland samples demonstrated elemental distributions for sulfur (correlated to overall cell thickness) and phosphorus (utilized as a marker for nuclei) to be typical of eukaryotic cells [27]. We examined the spatial distribution of Zn relative to phosphorus in mammary cells from lactating mice that had been recently suckled (Figure 2, Suckled). Visualization of the spatial distribution Zn pools in suckled mice represented a “basal” starting point to explore the initial accumulation and subsequent redistribution of intracellular Zn pools. We consistently detected Zn associated either directly with nuclei and/or in a distinct peri-nuclear compartment in recently suckled mice.

We next explored the spatial distribution of intracellular Zn pools in the mammary gland following Zn acquisition from maternal circulation during lactation. As can be seen in Figure 2 (Post-suckled), the lactating mammary epithelial cell accumulated significant Zn in preparation for its subsequent secretion into milk. Approximately 50% of the intracellular Zn was associated with a large, distinct nuclear/peri-nuclear pool, while the remaining Zn appeared to be concentrated in a few, large intracellular pools. These data suggest that Zn accumulates in large intracellular pools both in close approximation to and distal from the nucleus. This intracellular accumulation of Zn in mammary epithelial cells appears to occur in response to prolactin as mice injected with bromocriptine (which eliminated circulating prolactin) accumulated significantly less Zn compared with vehicle-treated mice. The minimal amount of Zn that was detected within the mammary epithelial cell remained distinctly associated with the nuclear/peri-nuclear compartment.

We have previously determined that prolactin stimulates Zn secretion from cultured mammary cells *in vitro* [23]. This suggests that the intracellular Zn storage pools we observed in the lactating mammary gland are redistributed for secretion in response to lactogenic cues. To explore effects of prolactin on cellular Zn redistribution *in vivo*, we injected mice with haloperidol which stimulated prolactin secretion ∼5-fold (675±427 ng/mL compared with 156±120 ng/mL in vehicle-treated mice). As indicated in Figure 2, haloperidol treatment clearly resulted in the spatial redistribution of intracellular Zn pools from the large, distinct intracellular Zn pools observed in post-suckled mice to numerous smaller vesicles which were distributed throughout the mammary epithelial cell. This did not appear to reflect increased Zn accumulation as the amount of Zn within the mammary epithelial cell from haloperidol-treated mice was actually lower that the amount of Zn in post-suckled, vehicle-treated controls. These observations are consistent with our previous determination that prolactin increases Zn secretion from mammary cells through a vesicular process [23].

**Figure 3:**
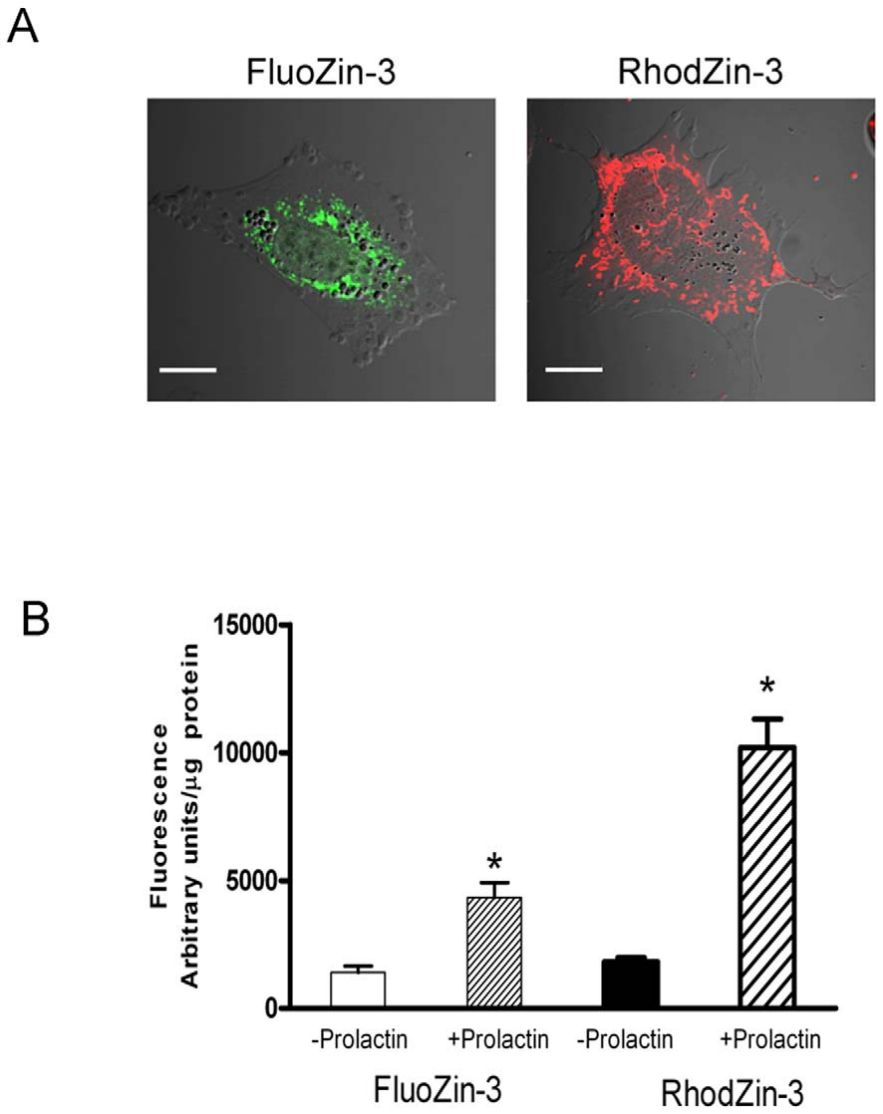
The lactogenic hormone prolactin increases vesicular and mitochondrial Zn pools in mammary cells. (A) Representative confocal images to illustrate vesicular and mitochondrial Zn pools detected using FluoZin-3 (green) and RhodZin-3 (red), respectively, in mammary cells (HC11). Scale bar (5 µm). (B) Mammary cells were treated with prolactin (1 µM) for 24 h and the accumulation of Zn in vesicular and mitochondrial Zn pools were detected by fluorometry using FluoZin-3 (green) and RhodZin-3 (red), respectively. Data represent mean fluorescence ± standard deviation, n = 6 samples/treatment. Asterisk indicates a significant effect of treatment (p<0.001).

Characterization of these intracellular Zn pools is critical to understanding how this process is regulated. The current limitations in XFM technology with respect to sub-cellular resolution required that we utilize cultured mammary epithelial cells to investigate these intracellular Zn pools more directly. Changes in the spatial distribution of Zn *in vivo* suggested that Zn is accumulated and sequestered in peri-nuclear as well as large, discrete intracellular pools. To explore the identity of these intracellular Zn pools, we hypothesized that mitochondria, ER, Golgi and/or lysosomes may provide sites for Zn accumulation. To test this hypothesis, we first visualized Zn pools using confocal microscopy. We capitalized on the specificity of RhodZin-3 and FluoZin-3, Zn-specific fluorophores which are specific for mitochondrial and “labile” Zn pools in vesicles to explore this hypothesis. Confocal micrographs of mammary epithelial cells *in vitro* clearly detected discrete Zn pools localized in peri-nuclear compartments including mitochondria and vesicles (Figure 3A). Consistent with our results *in vivo*, quantitative fluorometry in cultured mammary epithelial cells indicated that prolactin stimulates significant Zn accumulation into pools which appears to reflect increases in both mitochondria (∼10-fold increase) and vesicle (∼4-fold increase) Zn pools (Figure 3B). We interpreted this data to indicate that both mitochondria and vesicles are storage depots for Zn accumulation in mammary epithelial cells which may be redistributed to provide Zn for secretion.

**Figure 4:**
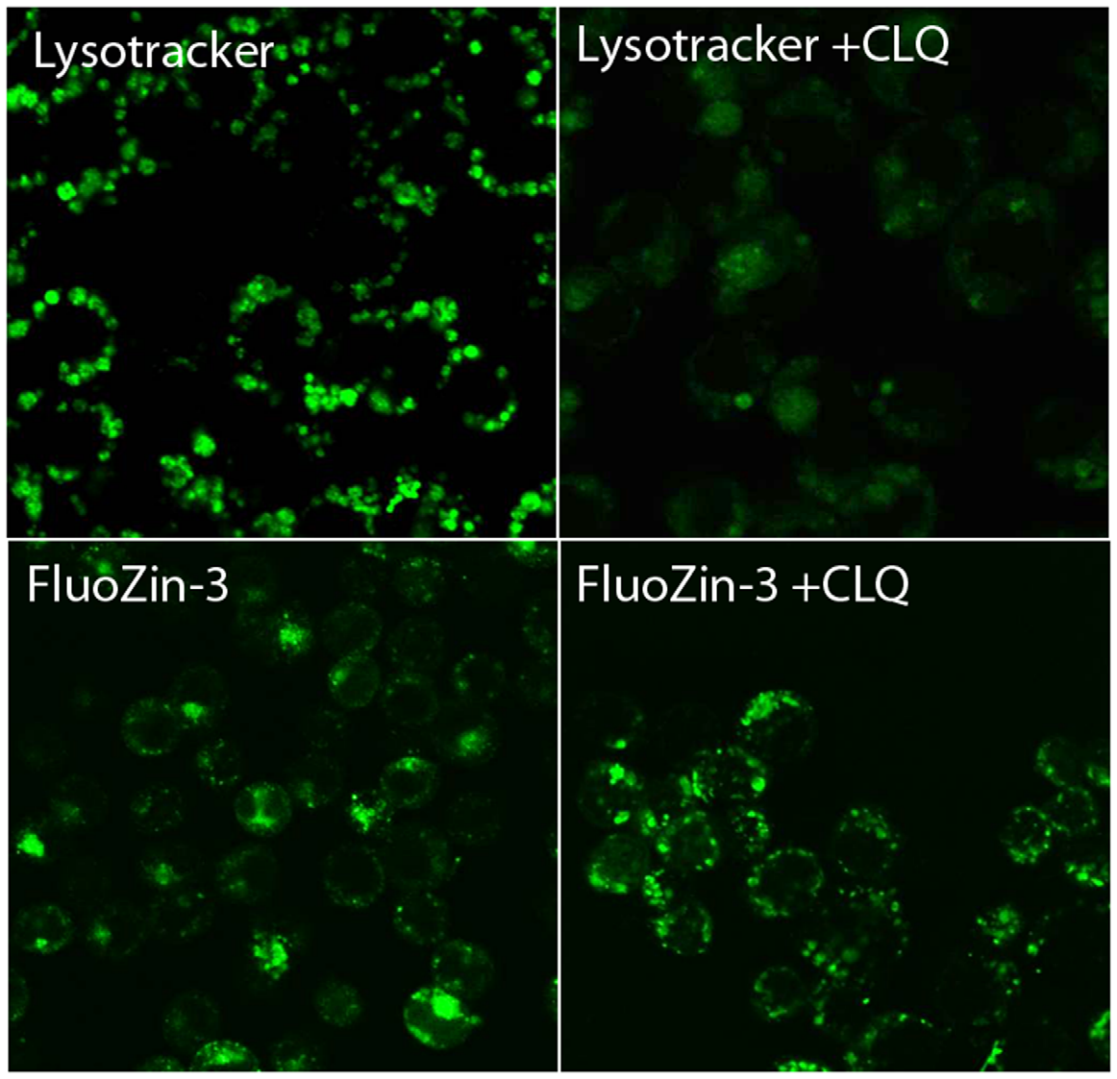
Labile Zn pools are not associated with lysosomes. Representative confocal micrographs of lysosomes detected with Lysotracker Green in untreated cells (Lysotracker) and cells treated with chloroquine diphosphate (Lysotracker +CLQ).. Images verify that chloroquine diphosphate disrupts lysosomes in HC11 cells. Representative confocal micrographs of “labile” Zn pools detected using FluoZin-3 in untreated cells (FluoZin-3) and cells treated with chloroquine diphosphate (FluoZin-3 +CLQ). Images illustrate that the distribution of “labile” Zn pools in mammary cells is not affected by lysosomal disruption.

**Figure 5:**
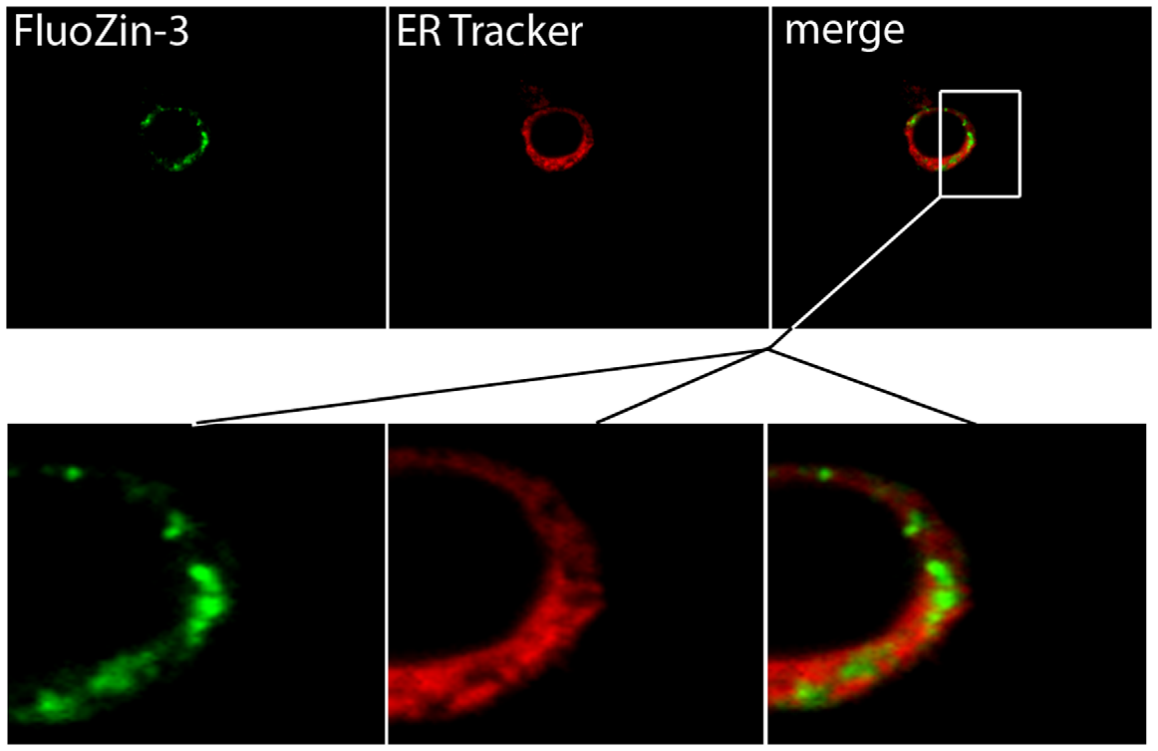
Labile Zn pools are associated with a distinct compartment directly proximal to the endoplasmic reticulum. Representative confocal micrographs of mammary epithelial cells treated with FluoZin-3 (green) and ER Tracker (red) which illustrate that “labile” Zn pools do not exist within the endoplasmic reticulum and are, in fact, associated with a distinct compartment proximal to the endoplasmic reticulum (merge).

**Figure 6:**
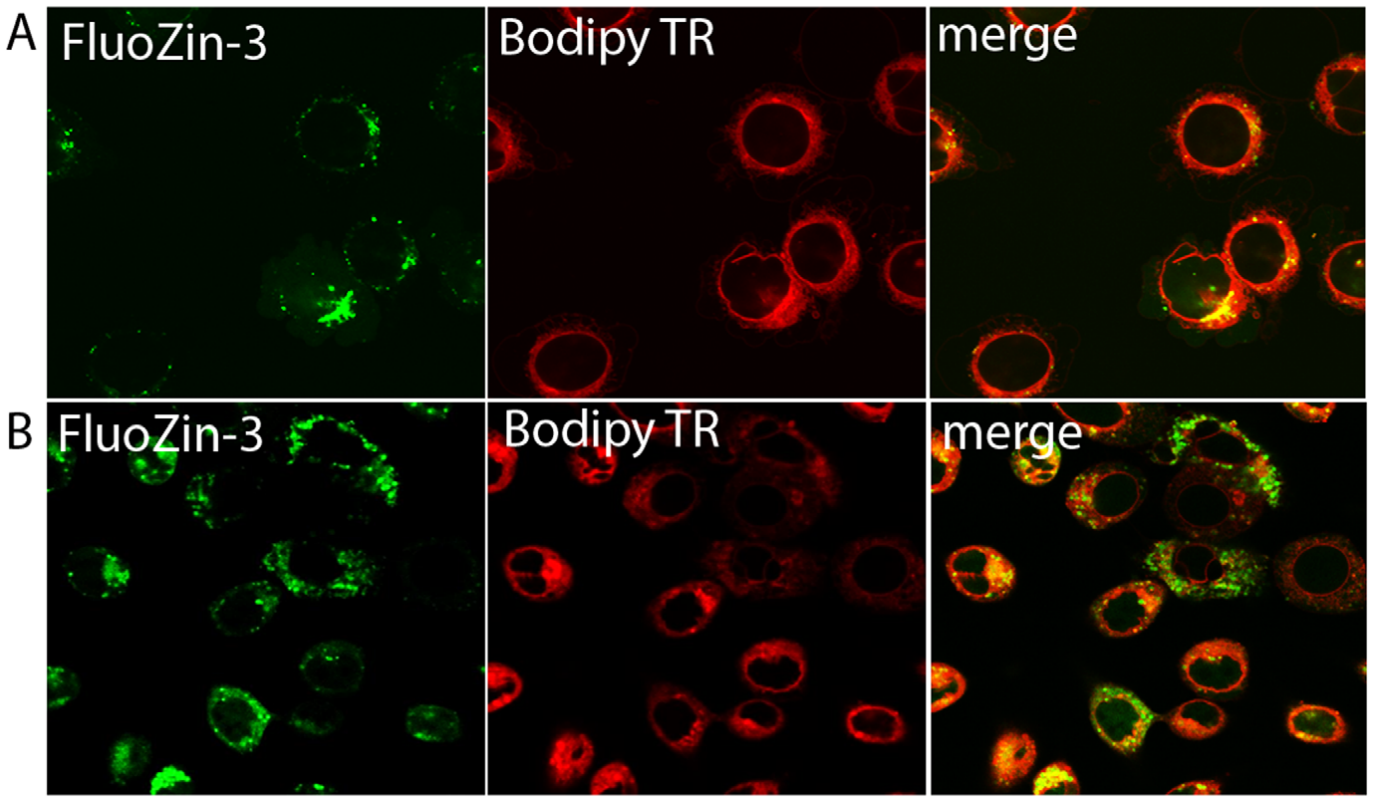
Labile Zn pools are partially associated with the Golgi apparatus. Representative confocal micrographs illustrate that FluoZin-3 (green) and Bodipy TR (red) are largely co-localized (merge, yellow) in non-secreting mammary epithelial cells (panel A). Mammary epithelial cells were treated with prolactin and cortisol for 24 h differentiate to a secreting phenotype (panel B). Confocal micrographs of FluoZin-3 (green) and Bodipy TR (red) in secreting mammary epithelial cells illustrate partial co-localization (merge, yellow) of “labile” Zn and the Golgi apparatus in hormonally treated cells. Moreover, distinct non-Golgi apparatus-associated Zn pools were detected in secreting cells.

**Figure 7:**
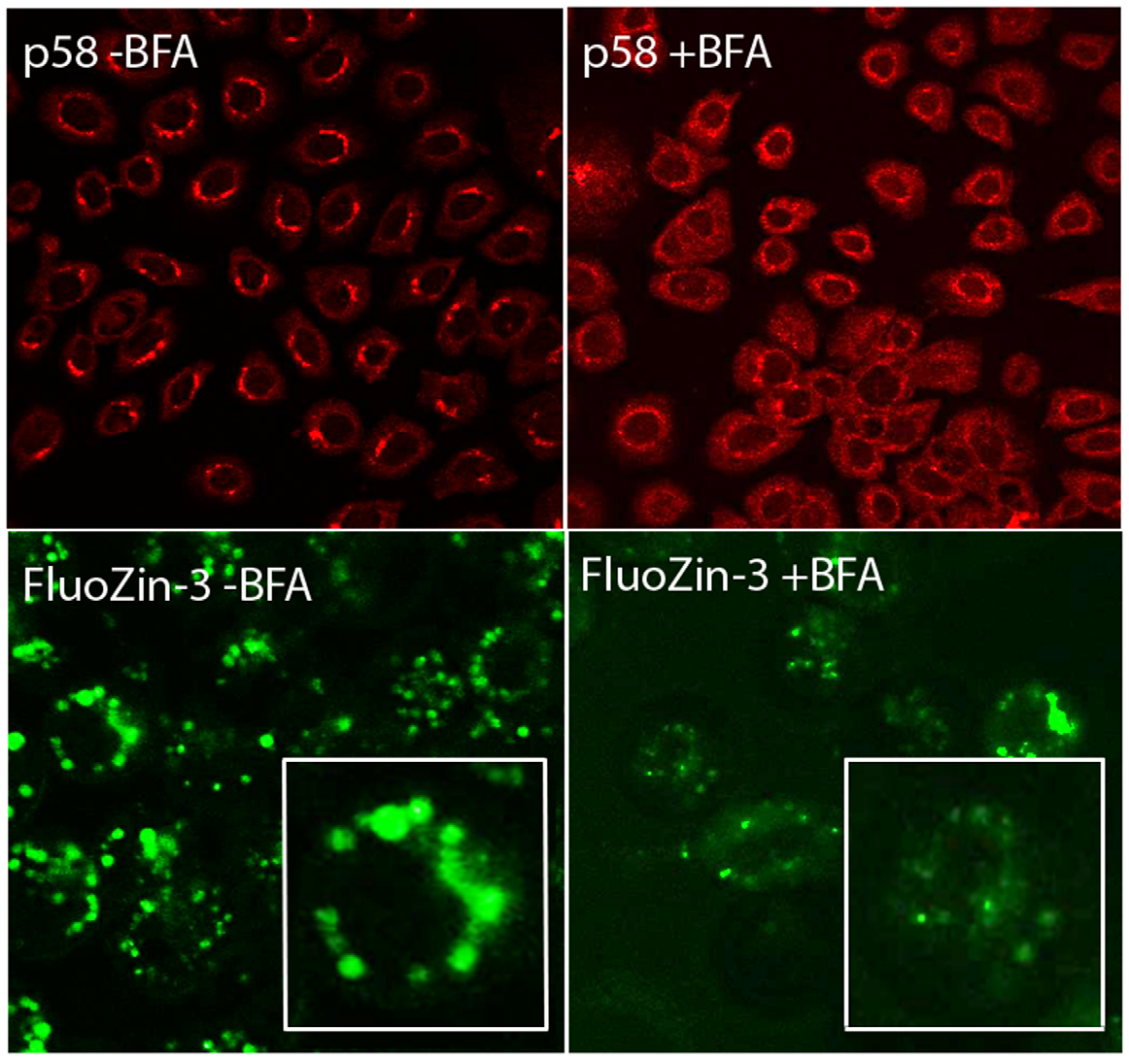
Labile Zn pools are disrupted by Brefledin A treatment. Representative confocal micrographs of p58 (Golgi apparatus marker) visualized with Alexa Fluor® 568-conjugated anti-mouse IgG in untreated (p58 −BFA) and Brefeldin A-treated (p58 +BFA) mammary epithelial cells. Images verify that Brefeldin A treatment disrupts the Golgi apparatus in mammary epithelial cells. Representative confocal micrographs of FluoZin-3 in untreated cells (FluoZin-3 −BFA) and cells treated with Brefeldin A (FluoZin-3 +BFA). Images illustrate that the distribution of “labile” Zn pools is diffuse by Brefeldin A treatment (inset) indicating that “labile” Zn is associated with the Golgi apparatus.

To ascertain the identity of the vesicular pool(s), the spatial distribution of intracellular Zn pools in mammary cells was visualized by confocal microscopy. We verified that chloroquine pre-treatment disrupted the lysosomes as illustrated by Lysotracker Green staining (Figure 4A and 4B); however, the spatial distribution of FluoZin-3 fluorescence was not disrupted by chloroquine pre-treatment (Figure 4C and 4D). Identical results were obtained in cells differentiated to a secreting phenotype (data not shown), thus eliminating the lysosomes as a Zn-rich compartment in mammary epithelial cells. We next attempted to co-localize intracellular Zn pools with the endoplasmic reticulum (ER) using ER Tracker. As illustrated in Figure 5, no co-localization between the ER and FluoZin-3 was detected eliminating the ER as a Zn-rich compartment in mammary cells. Identical results were obtained in cells differentiated to a secreting phenotype (data not shown). In fact, intracellular Zn pools were associated with a distinct compartment immediately proximal to the ER (Figure 5, inset). In contrast, we co-localized FluoZin-3 with Bodipy TR which stains sphingolipids and is routinely used to visualize the Golgi apparatus (Figure 6). This suggested that the Golgi apparatus was a site of Zn accumulation in mammary epithelial cells. To verify the association of “labile” Zn pools with the Golgi apparatus, pre-treatment with Brefeldin A effectively disrupted the Golgi apparatus and clearly affected the spatial distribution of “labile” Zn in mammary cells (Figure 7), confirming that the Golgi apparatus contains a Zn-rich intracellular pool. Interestingly, cells differentiated to a secreting phenotype with prolactin had Zn pools that were partially associated with the Golgi apparatus and partially another distinct non-ER and non-lysosome but vesicular compartment. These results are consistent with our observations using XFM *in vivo* and our previous determination that Zn accumulates into ZnT2-associated exocytotic vesicles for secretion from the mammary cell in response to prolactin [23].

**Figure 8:**
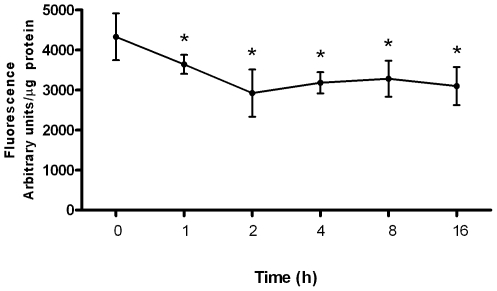
Prolactin stimulates the mobilization of vesicular Zn pools in secreting mammary cells. Mammary cells were treated with prolactin for 24 h to differentiate to a secreting phenotype. Following differentiation, cells were stimulated with prolactin and changes in “labile” Zn pools were quantified by fluorometry using FluoZin-3. Data represent mean fluorescence ± standard deviation, n = 6–8 samples/treatment. Asterisk denotes a significant effect of prolactin on FluoZin-3 fluorescence, p<0.001.

To quantify this redistribution *in vitro*, we differentiated cells to a secreting phenotype thereby permitting the accumulation of intracellular Zn pools (i.e., into mitochondria and the Golgi apparatus). We then treated secreting cells with prolactin to determine if we could mobilize these Zn pools assessed by changes in RhodZin-3 and FluoZin-3 fluorescence. We did not detect a reduction in mitochondrial Zn pools using RhodZin-3 (data not shown). In contrast, we detected a significant decline (p<0.001) in vesicular Zn pools using FluoZin-3 in response to prolactin stimulation (Figure 8) consistent with our observations *in vivo*. Together our data suggest that Zn accumulates in mitochondria and the Golgi apparatus in a hormonally-dependent manner, after which Golgi apparatus Zn pools are redistributed in response to prolactin for secretion. In contrast, while mitochondrial Zn pools do expand, these pools do not appear to contribute significantly to Zn secretion. Importantly, our data demonstrate this redistribution occurs both *in vitro* and *in vivo*.

## Discussion

The secretion of an extraordinary amount of Zn into milk during lactation is critical to providing optimal nutrition for maximizing the health and development of the nursing offspring. The process by which the mammary gland takes up such an astonishing amount of Zn from maternal circulation, and then ultimately secretes it into milk must integrate Zn import, accumulation and secretion mechanisms [28], [29], [30]. Moreover, consistent with the regulation of lactation and the production and/or secretion of other milk components, the coordination of Zn secretion processes must be regulated through complex hormone-mediated events. X-ray fluorescence microscopy is a powerful tool permitting the visualization and quantification of metal pools in tissue sections [27]. The use of XFM in this study allowed us to directly visualize the accumulation and redistribution of discrete intracellular Zn pools in the lactating mammary gland. Our data illustrated that Zn imported from maternal circulation is not likely transported directly across the mammary gland and into milk but is instead accumulated in intracellular compartments within the mammary cell. This novel vesicular accumulation/redistribution paradigm likely permits tight control of the process of bulk Zn secretion. While we cannot yet identify these discrete intracellular compartments by hard XFM due to limited spatial resolution, our data from cultured mammary cells suggest that several distinct cellular compartments may play a role in this process. A key finding from this study was the determination that Zn accumulates in large discrete pools associated with and/or proximal to the nucleus in mammary cells of lactating mice. Our data *in vivo* and *in vitro* clearly implicate mitochondria and the Golgi apparatus as sites of Zn accumulation in this cell type.

Mitochondria have a specific requirement for Zn and have been shown to accumulate Zn in neurons and prostate. As a result, it has been postulated that mitochondria serve as a mobilizable pool of Zn which is dependent upon cellular requirements [31]. In neurons, mitochondrial Zn accumulation is believed to occur through Ca transport mechanisms and is redistributed to a cytosolic pool in response to Ca-mediated signaling mechanisms [24]. Consistent with our previous study and observations in prostate mitochondria [32], [33], [34] and spermatogonia mitochondria [35], results from this study directly confirmed that mitochondria in cultured mammary epithelial cells accumulate Zn. Understanding how mitochondria accumulate Zn is of particular interest. The Zn transporter Zip8 is associated with mitochondria in lung epithelium [36]. Although directionality of Zn transport via Zip8 has not been substantiated, topology prediction suggests that it would transport Zn away from mitochondria and into the cytoplasm. In contrast, we have recently determined that one mechanism through which mitochondria in mammary epithelial cells directly accumulate Zn is through the Zn transporter ZnT2 (SLK, unpublished observations). Importantly, expression of ZnT2 is increased in response to the lactogenic hormone prolactin [19], [23], consistent with effects of prolactin on mitochondrial Zn accumulation. A key question that arises from this observation is, do mitochondrial Zn pools provide a mobilizable pool of Zn for secretion? In contrast to neurons, our data using RhodZin-3 as a mitochondrial Zn biosensor do not support the postulate that mitochondria serve as a rapid hormonally-regulated mobilizable Zn pool in mammary cells. Instead, we speculate that changes in mitochondrial Zn pools may perform regulatory functions to modulate the phenotype of the highly differentiated secretory epithelium during lactation. Studies are currently underway to test this hypothesis.

In addition to mitochondria, the endoplasmic reticulum and Golgi apparatus reside in close approximation to the nucleus suggesting that these compartments may sequester Zn prior to secretion into milk. In fact, a recent study using XFM detected a peri-nuclear Zn pool in keratinocytes which was postulated to reside in the ER or Golgi apparatus [37]. Our studies provide direct evidence that the Golgi apparatus but not the ER provides a Zn-rich pool in mammary epithelial cells. Several Zn transporters have been postulated to transport Zn into the Golgi apparatus including ZnT4-ZnT7 [38], [39], [40]. Our documentation of a Zn-rich and mobilizable pool in the Golgi apparatus of mammary cells is consistent with observations of reduced Zn content in milk of ZnT4^−/−^ (*lethal milk*) mice [41] and suggest that ZnT4^−/−^ mice cannot expand the Zn pool in the Golgi apparatus to provide Zn for secretion into milk. However, Michalcyzk et al. noted that there was no obvious overlap between ZnT4 and Zinquin-stained vesicles in PMC42 cells [39] implicating ZnT4 in the accumulation of “non-labile” Zn pools as well. ZnT4 may not be uniquely responsible for this process. In addition to ZnT7 [40], [42], the yeast homologues to ZnT5 (Msc2p) [43] and ZnT6 (Zrg17) [44], [45] have all been localized to the ER and/or the Golgi apparatus and studies support a role for ER and/or Golgi apparatus Zn accumulation. Bioinformatic prediction suggests that ZnT5 and ZnT7 but not ZnT6 are expressed in the mammary gland [46] suggesting that ZnT5 and ZnT7 may also play a role in Zn sequestration in the ER and/or Golgi apparatus of the mammary epithelial cell as well. However, to our knowledge ZnT5-null [47] and ZnT7-null [48] mice do not have defects in Zn secretion from the mammary gland, suggesting that unlike ZnT4, ZnT5 and ZnT7 may not play major roles in this process. With that said, bioinformatic prediction must always be viewed with caution and followed up with empirical analysis; thus ZnT6 cannot be ruled out as a contributor to mammary gland Zn metabolism.

An interesting observation we made in this study is that once accumulated in the Golgi apparatus, intracellular Zn pools are then redistributed into smaller vesicles dispersed throughout the cell in response to subsequent prolactin stimulation *in vivo*. This is consistent with our previous data *in vitro* which indicate that the lactogenic hormone prolactin increases Zn uptake, Zn accumulation and Zn secretion in mammary cells [11], [18], [23]. Clearly the secretion of vesicularized Zn pools would provide a successful mechanism to export the large amount of Zn into milk that is required by the developing neonate. Furthermore, our visualization of vesicular Zn accumulation is consistent with the theory of zincosomes [49] which function as a reversible Zn storage compartment. A recent study using XFM determined that this vesicular Zn is found in complex with sulfur, histidine and oxygen and thus is technically not “free” [49]. Importantly, this binding allows for accumulation against a concentration gradient but is predicted to have a lower affinity for Zn than binding motif found in structural sites like Zn fingers (Cys_2_His_2_, Cys_3_His, Cys_4_), or in metallothionein (Cys_4_) [49]. Similarly, studies indicate that Zn in milk is found in loose complex with the small molecular weight ligand citrate [50]. Our detection of mobilizable Zn-rich pools in the Golgi apparatus is consistent with the concept that a Zn/citrate complex may be accumulated in the Golgi apparatus prior to secretion into milk through the exocytosis of Golgi apparatus-derived vesicles [5]. Alternatively, Zn may accumulate in a Golgi apparatus storage pool which is directly exported from the Golgi apparatus and subsequently taken up into secretory vesicles. In support of this latter postulate, two members of the Zip family of Zn transporters, Zip7 [51] and Zip9 [52] are associated with the Golgi apparatus, implicating them in Zn export from this organelle. This postulate supports our previous characterization of a role for ZnT2 in the accumulation of Zn into VAMP8-containing exocytotic vesicles prior to secretion from the cell [23]. Clearly, further studies are needed to determine if Zip7 and/or Zip9 facilitate Zn mobilization from the Golgi apparatus for import into ZnT2-associated secretory vesicles and if so, what the regulatory contribution of the Golgi apparatus is in mammary gland Zn secretion.

In summary, results from this study document for the first time that Zn transport in the mammary gland during lactation is regulated in a biphasic manner. First, Zn is accumulated into a peri-nuclear storage pool that includes mitochondria and the Golgi apparatus. Following subsequent lactogenic stimulation, Zn is redistributed into a vesicular pool for secretion from the mammary gland into milk. Our observations in mammary epithelial cells are consistent with that in prostate whereby autometallographic imaging illustrates that Zn is confined to vesicles associated with the apical (luminal) membrane [22], [53]. Together these data suggest that the large amount of Zn that is secreted into the two most Zn-rich biological fluids (milk and prostate fluid) may utilize similar processes which are clearly regulated through the integration of numerous Zn transporters. Further studies are needed to understand the complex integration and regulation of this process in highly specialized secretory tissues.

## Materials and Methods

### Ethics Statement

This study was approved by Central Biological Laboratory at the Pennsylvania State University, which is accredited by the American Association for the Accreditation of Laboratory Animal Care (AAALAC). All animal studies have been conducted according to Animal Welfare Act and the Public Health Service Policy. The animals were housed in pathogen-free units at The Pennsylvania State University, in compliance with IACUC regulations (IACUC #28141). Animals were maintained in a controlled environment which included filtered air and a 12 hour light/dark cycle. All animals had free access to food and water.

*Mice* –Virgin or lactating mice (lactation day 4–5) were obtained commercially (Charles River) and fed commercially available rodent chow *ad libitum*. To modulate circulating prolactin concentrations, mice (n = 5 mice/group) mice were injected with either bromocriptine (intraperitoneal, 0.4 mg/100 g body weight) in 20 mM tartaric acid/30% ethanol; haloperidol (subcutaneous, 1mg/kg body weight) in 20 mM tartaric acid/30% ethanol; or vehicle alone (20 mM tartaric acid/30%ethanol). Mice were killed by CO_2_ asphyxiation and blood drawn by cardiac puncture. Prolactin concentration was measured using a commercially available ELISA kit (Amersham Pharmacia Biotech).

### Tissue sections

Inguinal mammary glands were dissected from lactating mice and plunge-frozen in an ice-cold isopentane bath. Tissue sections (20 µm) were mounted intact on silicon nitride windows (Silson, Blisworth, U.K.) and thawed onto windows at room temperature. Sections were stored desiccated at room temperature until transported to Argonne National Laboratory for X-ray fluorescent microscopy.

### X-Ray Fluorescence Microscopy

Sections were imaged with the scanning x-ray microprobe at beamline 2-ID-E at the Advanced Photon Source (Argonne, IL). Undulator-generated x-rays of 10-keV incident energy were monochromatized with a single bounce Si 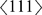 monochromator and focused to a measured spot size of 0.4×0.5 µm using Fresnel zone plate optics (X-radia, Concord, CA). Cells were raster-scanned in steps of 1.0 µm, and fluorescence spectra were collected for 1 s per pixel with a single-element silicon drift detector (Vortex-EX, SII Nanotechnology, CA). Quantitation and image-processing of the x-ray fluorescence (XRF) datasets was performed with MAPS software (25). Quantitation of elemental content was achieved by fitting XRF spectra at each pixel, and comparing against a calibration curve derived from measurements of thin-film standards NBS-1832 and NBS-1833 (National Bureau of Standards, Gaithersburg, MD).

### Cell culture

Mouse mammary epithelial cells (HC11) were a gift from Dr. Jeffery Rosen (Houston, Texas) and used with permission of Dr. Bernd Groner (Institute for Biomedical Research, Frankfurt, Germany). Cells were routinely maintained in a non-secreting phenotype by culturing in “growth medium” (RPMI 1640 supplemented with 10% fetal bovine serum, insulin (5 µg/mL), EGF (10 ng/mL) and gentamycin). Where indicated, cells were differentiated to a secreting phenotype in “secretion medium” consisting of serum-free RPMI 1640, supplemented with insulin (5 µg/mL), prolactin (PRL, 1 µg/mL) and cortisol (1 µM) for up to 48 h.

### Visualization and assessment of changes in zinc pools in mitochondria (RhodZin-3) and vesicles (FluoZin-3)

To first validate that RhodZin-3 and FluoZin-3 specifically stain mitochondria and vesicle Zn pools, respectively, cells were seeded onto glass coverslips and cultured overnight until ∼60% confluent. Cells were rinsed twice with PBS then loaded with RhodZin-3 AM or FluoZin-3 AM (1 µM in DMSO containing pluronic acid 127 to a final concentration of 0.02%; Invitrogen) following manufacturer's instructions in OptiMEM for 1 h at 37°C. Cells were briefly rinsed twice with PBS and washed with PBS for 30 min at 25°C with constant shaking. Images were collected from live cells using a FV-1000 confocal microscope.

To quantify Zn accumulation in mitochondria and vesicles, cells were cultured until ∼80% confluence in 96-well optical bottom black-sided plates and differentiated to a secreting phenotype for 48 h were noted. Cells were rinsed twice with PBS then loaded with RhodZin-3 AM or FluoZin-3 AM as described above. Fluorescence of RhodZin-3 AM (excitation 550 nm, emission. 575 nm) and FluoZin-3 (excitation 494 nm, emission 516 nm) was measured at 25°C using FLUOstar OPTIMA plate reader (BMG Labtech) spectrofluorimeter with FLUOstar OPTIMA software version 1.32R2. Cellular protein concentration was determined by the Bradford assay (Pierce) and fluorescence measurements were normalized to total protein concentration. To determine if Zn is redistributed from mitochondria and vesicles in response to prolactin, cells were cultured until ∼80% confluence in 96-well optical bottom black-sided plates and differentiated to a secreting phenotype for 48 h then subsequently treated with prolactin for up to 16 h. Mitochondrial and vesicular Zn pools were quantified as described above.

Next, to identify the vesicular compartments that accumulate Zn, cells were cultured on glass coverslips to ∼80% confluency and pre-treated with chloroquine disphosphate salt (Sigma, 100uM for 180 min) to disrupt lysosomes. Control experiments using Lysotracker Green (Molecular Probes, 1∶1000 dilution for 30 mins at 37°C) first verified that chloroquine diphosphate treatment disrupted the lysosomes by confocal imaging. Cells pre-treated with chloriquinone were secondarily treated with FluoZin-3 AM as described above to visualize “labile” Zn pools and images of “labile” Zn pools were collected. To determine if the Golgi apparatus accumulated and/or redistributed Zn pools, cells were first pre-treated with Brefeldin A (Sigma, BFA, 1 ug/ml for 120 min) to disrupt the Golgi apparatus. To verify that BFA treatment disrupted the Golgi apparatus, subcellular localization of the Golgi protein p58 was determined in cells pre-treated with BFA and compared with untreated cells. To determine the sub-cellular localization of the p58 protein, HC11 cells plated on to glass coverslips, were fixed in phosphate buffered-paraformaldehyde (4%; w/v), pH 7.4, for 10 min, washed in PBS, and permeabilized with Triton X-100 (0.5% in PBS) for 5 min. Non-specific binding was blocked with 5% goat serum in PBS for 60 min followed by incubation with p58 antibody (Sigma, 1∶100 dilution in 5% goat serum/1% BSA/0.5% Triton-X100) for 60 minutes. After extensive washing with PBS, p58 antibody was detected with Alexa Fluor® 568-conjugated anti-mouse IgG (1 µg/ml; Invitrogen) for 20 min at room temperature shielded from light. Cells were washed, mounted in ProLong Gold (Invitrogen), and sealed with nail polish. Cells pre-treated with Brefeldin A were secondarily treated with FluoZin-3 AM as described above to visualize “labile” Zn pools and images of “labile” Zn pools were collected. To verify that the Golgi apparatus accumulated Zn pools, cells were cultured on glass coverslips to ∼80% confluency, rinsed twice with PBS then loaded with BODIPY TR dye-labeled sphingolipid (5mg in sterile, deionized water to a final concentration of 0.5mM sphingolipid, Invitrogen) following manufacturer's instructions in OptiMEM for 30 minutes at 4°C. Cells were subsequently treated with FluoZin-3 AM to visualize “labile” zinc pools, and washed with PBS for 30 min at 25°C with constant shaking. Images were collected from live cells as described above. To determine if the endoplasmic reticulum accumulated Zn pools, cells were cultured on glass coverslips to ∼80% confluency. Cells were rinsed twice with PBS then loaded with ER-Tracker Dye (100 µg in DMSO to a final concentration of 90.9% w/v; Invitrogen) following manufacturer's instructions in OptiMEM for 60 minutes at 37°C. Cells were subsequently treated with FluoZin-3 AM to visualize “labile” zinc pools, and washed with PBS for 30 min at 25°C with constant shaking. Images were collected from live cells using as described above.

### Statistical analysis

Results are presented as mean ± standard deviation. A minimum of at least two independent experiments were conducted with sample sizes as indicated. Statistical comparisons were performed using Student's *t*-test (Prism Graph Pad, Berkeley, CA) and a significant difference was demonstrated at p<0.05.
